# Cranial and facial inter-landmark distances and tissue depth dataset from computed tomography scans of 388 living persons

**DOI:** 10.1016/j.dib.2022.108334

**Published:** 2022-05-29

**Authors:** Terrie L. Simmons-Ehrhardt, Connie L. Parks, Keith L. Monson

**Affiliations:** a Visiting Scientist Program, Federal Bureau of Investigation, Laboratory Division, 2501 Investigation Parkway, Quantico, VA 22135; b School of World Studies, Virginia Commonwealth University, 312 N. Shafer St, Richmond, VA 23284; c Federal Bureau of Investigation, Laboratory Division, 2501 Investigation Parkway, Quantico, VA 22135

**Keywords:** Forensic anthropology population data, Craniofacial landmarks, Cranial measurements, Facial measurements, Facial soft tissue thickness, CT model, Facial approximation, Craniofacial identification

## Abstract

Computed tomography (CT) scans of 388 living adults of both sexes were collected from four self-identified ancestry groups from the United States (African, Asian, European, and Hispanic). Scans were acquired from multiple institutions and under a variety of scanning protocols. Scans were used to produce 3D bone and soft tissue models, from which were derived cranial and facial inter-landmark distances (ILDs) and soft tissue depth measurements. Similar measurements were made on 3D facial approximations produced by ReFace software. 3D models and all measurements were obtained using Mimics^R^ software. These measurements are useful for facial approximations of unidentified decedents and for investigations into human variation between and among ancestry groups and sexes.

## Graphical abstract

**Figure:**
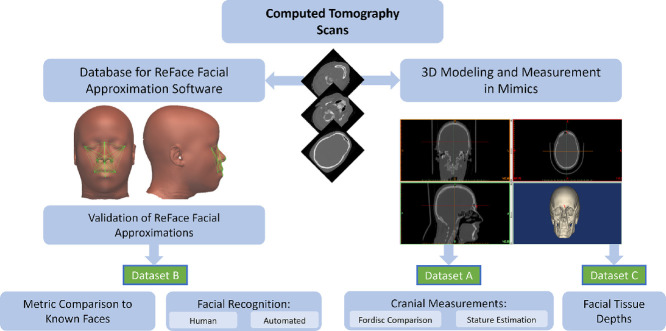

**Specifications Table**

**Table:** 

Subject	Forensic Medicine
Specific subject area	Cranio-facial measurements derived from CT models of living humans and from their 3D facial approximations obtained using ReFace software
Type of data	Tables
How the data were acquired	Mimics^R^ software (Materialise, Ann Arbor, MI) was used to produce 3D models from CT scans and to measure inter-landmark distances (ILD) and soft tissue depths on the models. ILDs on 3D facial approximations and known faces were measured using a custom interface that allowed automated extraction of homologous landmark coordinates.
Data format	Raw
Description of data collection	Adult American individuals undergoing CT scans for medical reasons were given the option to provide their scan for a research study. All donors provided informed consent via a form approved by the institutional review boards of the participating medical institutions. Data collected at the time of the scan included age, ancestry, sex, height, and weight. Participants were categorized into four descent groups based on self-identification: African, Asian, European, and Hispanic.
Data source location	• Federal Bureau of Investigation, Laboratory Division • 2501 Investigation Parkway, Quantico, VA 22135 • U.S.A.
Data accessibility	Repository name: Mendeley Data Data identification number: DOI: 10.17632/byr94xy7mv.1 DOI: 10.17632/cnc2j4h26b.1 DOI: 10.17632/pss59h29nb.1 Direct URL to data: https://doi.org/10.17632/byr94xy7mv.1 https://doi.org/10.17632/cnc2j4h26b.1 https://doi.org/10.17632/pss59h29nb.1
Related research article	T.L. Simmons-Ehrhardt, T. Flint, C.P. Saunders, K.L. Monson, Quantitative accuracy and 3D biometric matching of 388 statistically estimated facial approximations of live subjects, Forensic Img 21 (2020) 200377. https://doi.org/10.1016/j.fri.2020.200377 C.L. Parks, A.H. Richard, K.L. Monson, Preliminary assessment of facial soft tissue thickness utilizing three-dimensional computed tomography models of living individuals, Forensic Sci. Int. 237 (2014) 146.e1–146.e10. doi:10.1016/j.forsciint.2013.12.043

**Value of the Data**

• These data are useful for informing skull to face relationships for forensic identification methods: facial approximations and craniofacial superimposition of unidentified decedents and for investigations into human craniofacial variation
• Potential users of these datasets include forensic anthropologists, biological anthropologists, forensic artists, craniofacial identification experts, and plastic and reconstructive surgeons.
• The cranial data may be used for comparison with other populations and other craniometric methods for estimating sex and ancestry. Facial inter-landmark distances and tissue depths can be used to inform and evaluate facial approximations and craniofacial superimposition.
• The tissue depth dataset may be the first and largest multi-ancestry American sample of tissue depths where all data derive from a single study.

## Data Description

1. Cranial ILDs on CT-derived models (Dataset A)

Cranial ILD measurements derived from 3D bone models of CT scans collected from 287 living subjects in the U.S.A. are provided in dataset A [1,2]. The dataset comprises both sexes and three ancestry groups: African female (n=56), African male (n=45), Asian female (n=46), Asian male (n=47), European female (n=46), European male (n=47). Data for each group are found in worksheets AFRF, AFRM, ASNF, ASNM, EURF, EURM, respectively, where the final F or M in worksheet name designates sex (also designated in column Sex). Each line presents data for one individual.

Most ILD measurements (Table 1) were defined by standard craniometric definitions used in Fordisc [3]. Practical constraints of CT imaging obliged slight redefinition of measurements for OBB, OBH, and XCB from what is used for traditional caliper measurements to accommodate the positioning of landmarks from which to derive these measurements. Orbital breadth (OBB), normally demarcated by left and right dacryon, was redefined by left and right maxillofrontale [4], which was necessitated by limited discernability of dacryon in many scans. Orbital height (OBH), was referenced by identifying the midpoint (MsorL/R) of the superior orbital border and positioning IorL/R on the inferior orbital border to generate a line perpendicular to OBB. Absent the physical feedback provided by a caliper on a skull, maximum cranial breadth (XCB) was redefined by left and right euryon (Eu), the most lateral points of the skull being visualized in orthogonal views. A measurement template generated in the simulation module of the Mimics^R^ software provided the 3D distance between specified landmarks.

2. *Facial ILDs on CT-derived models and on ReFace approximations (Dataset B)*

**Table 1:** Cranial Interlandmark Distances (ILDs) reported.

Interlandmark Distances (ILDs)	Abbr.	Defined by
Biauricular breadth	AUB	au-au
Basion-bregma height	BBH	ba-br
Cranial base length	BNL	ba-n
Basion-prosthion length	BPL	ba-pr
Frontal chord	FRC	n-br
Maximum cranial length	GOL	g-op
Maxillo-alveolar breadth	MAB	ecm-ecm
Nasal breadth	NLB	al-al
Nasal height	NLH	ns-n (left and right)
Orbital breadth	OBB*	mf-ec (left and right)
Orbital height	OBH*	msor-ior (left and right)
Occipital chord	OCC	l-o
Parietal chord	PAC	br-l
Upper facial height	UFHT	n-pr
Minimum frontal breadth	WFB	ft-ft
Maximum cranial breadth	XCB *	eu-eu
Bizygomatic breadth	ZYB	zy-zy

⁎ Text describes use of modified landmarks

In dataset B [5], 66 facial ILDs (Table 2) were defined by 12 standard anthropometric landmarks [6,7]. The CT database comprised both sexes and four ancestry groups (n = 388): African female (n=50), African male (n=48), Asian female (n=48), Asian male (n=47), European female (n=49), European male (n=48), Hispanic female (n=49), and Hispanic male (n=49). Data for each group are found in worksheets afrf, afrm, asnf, asnm, eurf, eurm, hisf, hism, respectively, where the final F or M designates sex (also listed in column Sex). The suffix "_k" designates measurements collected from CT-derived (k, known) models, while the suffix "_r" designates measurements collected from ReFace approximations (r, ReFace) models. Each column presents data for one individual.

3. *Tissue depths on CT-derived facial models (Dataset C)*

**Table 2:** Facial landmarks that define the reported ILDs.

Name	Abbreviation
sellion	se
exocanthion	exL, exR
pronasale	prn
alar curvature	acL, acR
subnasale	sn
labiale superior	ls
labiale inferior	li
stomion	sto
cheilion	chL, chR

Dataset C comprises soft tissue depths measured manually on CT-derived facial models [8,9]. Both sexes and four ancestry groups are represented, African female (n=50), African male (n=48), Asian female (n=48), Asian male (n=47), European female (n=49), European male (n=48), Hispanic female (n=49), and Hispanic male (n=49). The dataset includes measurements at 25 tissue depth locations defined by 14 mid-sagittal and 11 bilateral facial landmark pairs [10] on soft tissue 3D models derived from CT scans of 388 living subjects in the U.S.A. (Table 3).

**Table 3:** Facial landmarks that define the reported tissue depths.

Mid-sagittal Landmarks		Bilateral Landmarks	
g-g'	Glabella	acp-acp'	Alare curvature point
gn-gn'	Gnathion	go-go'	Gonion
li-li'	Labrale inferius	iC-iC'	Infra canine
ls-ls'	Labrale superius	iM^2^-iM^2^'	Infra M_2_
m-m'	Menton	mio-mio'	Mid-infraorbital
mls-mls'	Mentolabial sulcus	mmb'mmb'	Mid-mandibular border
mn-mn'	Mid-nasal	mr-mr'	Mid-ramus
mp-mp'	Mid-philtrum	mso-mso'	Mid-supraorbital
n-n'	Nasion	sC-sC'	Supra canine
op-op'	Opisthocranion	sM^2−^sM^2^'	Supra M^2^
pg-pg'	Pogonion	zy-zy'	Zygion
rhi-rhi'	Rhinion		
sn-sn'	Subnasale		
v-v'	Vertex		

Data for all groups are found in worksheet TD ReFace (tissue depth, ReFace). Each line presents data for one individual. Where the prefix AFR, ASN, CAU, HIS designates group affiliation and column B designates sex. Summary statistics are included at the end of worksheet TD ReFace, in worksheet Descriptives, and for European (CAU) males and females, in the worksheets so named.

## Experimental Design, Materials and Methods

Adult CT scans were collected between 2003-2009 by GE Global Research for initial use as a reference database for ReFace [11]. Collection and intended use of these anonymized data were approved by the institutional research boards of the partner medical institutions, and each subject signed an informed consent agreement. The CT scans were acquired from multiple institutions and were collected under a variety of scanning protocols, with slice thicknesses ranging from 0.98 mm to 6.00 mm, slice increments ranging from 0.10 mm to 5.00 mm, pixel size ranging from 0.449 mm to 0.586 mm, and three X-Y image resolutions [8]. Scans with 6.00 mm slice thickness had 2.90-3.00 mm slice increment, resulting in high overlap of slices and higher resolution images and models. As these CT scans were collected for medical purposes, there was no control over CT protocols (slice thickness and slice increment); however, the scans collected were deemed suitable for inclusion in ReFace by the software developers at GE.

CT scans originally collected for ReFace were segmented outside of ReFace into skull and face models in Mimics to collect and evaluate cranial measurements for comparison to an existing database of dry skull measurements in the Forensic Data Bank [12] and to collect facial soft tissue thicknesses for comparison to existing studies and to supplement facial soft tissue data of modern Americans available for facial approximation practitioners [10]. 3D skull and face models were generated in Mimics^R^ v. 11 and v. 12.0 by segmenting two masks using the default software bone/soft tissue threshold of 226 Hounsfield units (bone values were 226 and higher, soft tissue values were less than 226). Individual segmentations were manually edited as needed to remove burst artifacts and vertebrae before converting to 3D surface models using the Optimal reconstruction setting. The simulation module of Mimics was used to generate one template to place landmarks on the digital 3D skulls for cranial measurements [2], and a second template for the collection of facial soft tissue depths. Facial ILDs were compared between ReFace generated approximations and known faces within the ReFace software using a custom auxiliary interface built by GE that allowed for the placement of facial landmarks on one known index face per sex/ancestry group and automatic collection of the landmark coordinates for other known faces and approximations in that group.

**Figure 1:**
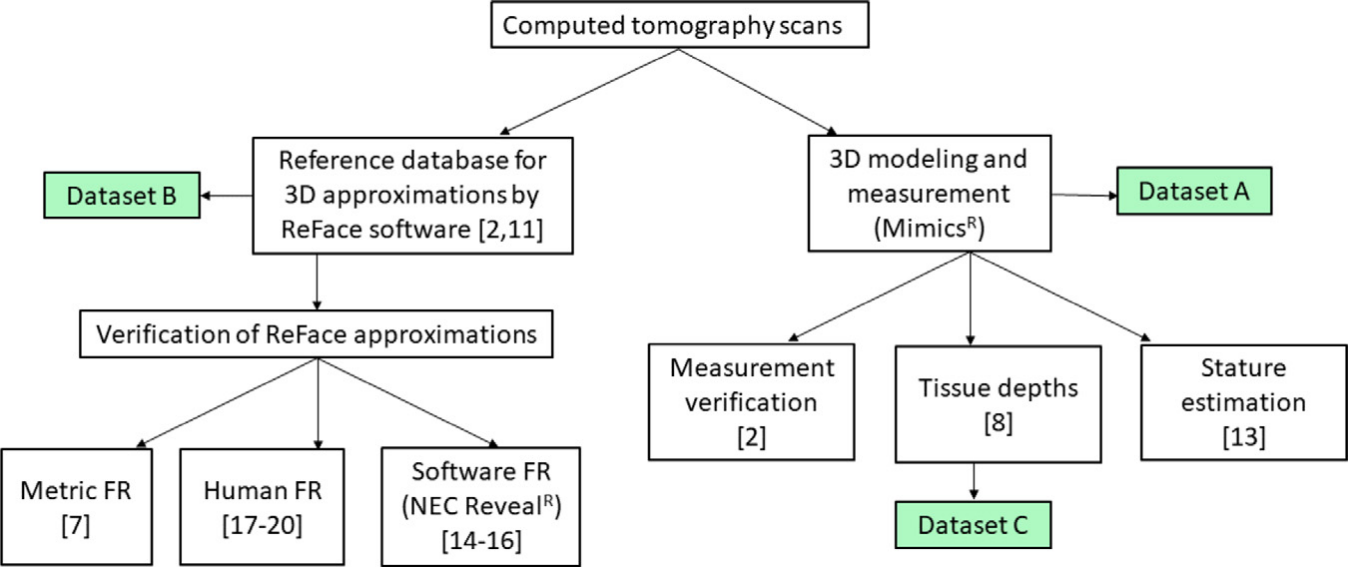
Experimental design, showing the source of datasets A, B, C and publications arising from each subtopic.

The overall design of the research program that validated the three datasets presented herein is outlined in Figure 1. The 3D models created from cranial CT scans of the 388 individuals were used as the reference database for verification of precision and accuracy of cranial measurements [2], to measure tissue depths [8], and to infer stature from cranial measurements [13]. CT-derived facial models and ReFace facial approximations were placed among the top candidates within a database of facial images, whether using a metric approach based on ILDs [7], commercial facial recognition software [14], [15], [16], [17], or by human recognition [18], [19], [20]. Three studies reported averaged ILDs and tissue depths from 3D models of cranial CT scans of living subjects or from their ReFace facial approximations [2,^7^,8]. This communication reports the ILDs and tissue depths measured for each subject upon which the previously reported averages were based. The corresponding facial images cannot be shared due to privacy considerations.

## Ethics Statements

Individuals undergoing CT scans for medical reasons were given the option to provide their scan for this research study. All donors provided informed consent via a form approved by the institutional review boards of the participating medical institutions.

## CRediT Author Statement

**Terrie L. Simmons-Ehrhardt:** Conceptualization, Methodology, Investigation, Data curation, Writing – review & editing; **Connie L. Parks:** Conceptualization, Methodology, Investigation, Data curation; **Keith L. Monson:** Supervision, Project administration, Resources, Conceptualization, Methodology, Data curation, Writing – original draft preparation.

## Declaration of Competing interest

The authors declare that they have no known competing financial interests or personal relationships that could have appeared to influence the work reported in this paper.

## Data Availability

Tissue depths on CT facial models and on ReFace approximations (Original data) (Mendeley Data).
Facial Interlandmark distances on CT soft tissue models of 388 living persons (Original data) (Mendeley Data).
Cranial interlandmark distances from CT bone models of 330 living persons (Original data) (Mendeley Data).
